# Genome Sequence of Novel Human Parechovirus Type 17

**DOI:** 10.1128/genomeA.01649-16

**Published:** 2017-02-23

**Authors:** Sindy Böttcher, Patrick E. Obermeier, Sabine Diedrich, Yolande Kaboré, Rossella D’Alfonso, Herbert Pfister, Rolf Kaiser, Veronica Di Cristanziano

**Affiliations:** a National Reference Centre for Poliomyelitis and Enteroviruses, Robert Koch Institute, Berlin, Germany; b Centre Don Orione pour Handicapés Physiques, Bonoua, Côte d’Ivoire; c Department of Systems Medicine, University of Rome Tor Vergata, Rome, Italy; d Institute of Virology, University of Cologne, Cologne, Germany

## Abstract

Human parechoviruses (HPeV) circulate worldwide, causing a broad variety of symptoms, preferentially in early childhood. We report here the nearly complete genome sequence of a novel HPeV type, consisting of 7,062 nucleotides and encoding 2,179 amino acids. M36/CI/2014 was taxonomically classified as HPeV-17 by the picornavirus study group.

## GENOME ANNOUNCEMENT

Human parechoviruses (HPeVs) are small nonenveloped single-stranded RNA viruses belonging to the genus *Parechovirus* within the family *Picornaviridae*. The genomes are of positive polarity and comprise ~7,400 nucleotides (nt), including an open reading frame flanked by a 5′ and 3′ untranslated region (UTR). The polyprotein is cleaved by viral proteases into 10 structural and nonstructural viral proteins. Currently, 16 different types of HPeV have been designated based on the VP1 capsid protein sequence (1) (http://www.picornaviridae.com). Moreover, one new type has been proposed that did not cluster with any of the 16 types (2). HPeVs are associated with a broad spectrum of diseases, including gastroenteritis, respiratory diseases, meningitis/encephalitis, acute flaccid paralysis, and neonatal sepsis (3). Nevertheless, the majority of subjects with HPeV infections may remain asymptomatic (4). We have recently reported a hitherto-unassigned HPeV strain (M36/CI/2014) that was identified in a stool sample from a healthy 9-month-old female from Côte d’Ivoire (5). To further analyze this strain, we have amplified and sequenced nearly the entire genome.

Based on parechovirus sequences available in GenBank, primers targeting the 5′ UTR (6) and 3D^pol^ region were used. The nearly entire genome was amplified using the PrimeScript one-step reverse transcription-PCR (RT-PCR) kit (TaKaRa Bio, Inc., Nojihigashi, Japan), and the 3′ UTR was amplified by one-step RT-PCR using 3′ rapid amplification of cDNA ends (3′RACE). Amplification of the complete 5′ UTR failed by using different 5′RACE kits. Therefore, 275 nt of the 5′ UTR of the genome is missing compared to the HPeV-1 prototype Harris (accession no. L02971). Sequencing was performed using a primer walking method directly on the PCR products after ExoSAP (Affymetrix, Santa Clara, CA, USA) treatment using BigDye Terminator 3.1 (Applied Biosytems, Foster City, CA, USA).

The nearly entire genome of M36/CI/2014 consists of 7,062 nt, excluding the 3′-poly(A) tail. It contains 433 nt of the 5′ UTR, an open reading frame (ORF) that is 6,540 nt in length encoding 2,179 amino acids (aa), and a 3′ UTR of 89 nt. BLAST search of the complete genome sequence revealed low nucleotide identity (79 to 82%) to HPeV reference genomes available in GenBank (HPeV-1, accession no. L02971; HPeV-2, accession no. AF055846; HPeV-3, accession no. AB084913; HPeV-4, accession no. DQ315670; HPeV-5, accession no. HQ696576; HPeV-6, accession no. HQ696577; HPeV-7, accession no. EU556224; and HPeV-8, accession no. EU716175). Although one BLAST hit reached 86% (accession no. GQ183020), phylogenetic analysis using the complete genome of M36/CI/2014 and 30 other HPeV genomes available in GenBank clearly showed that M36/CI/2014 is distinct from the known HPeV types.

### Accession number(s).

The virus genome sequence described here has been deposited in the GenBank database under the strain name M36/CI/2014 under the GenBank accession no. KT319121.
